# Organizational Factors and Burnout of Perioperative Nurses

**DOI:** 10.2174/1745017901814010132

**Published:** 2018-05-31

**Authors:** Amalia Sillero, Adelaida Zabalegui

**Affiliations:** 1 Department of Surgical Area, Hospital de la Santa Creu i Sant Pau, Barcelona, Spain; 2 Deputy Director of Nursing Research and Education, Hospital Clinic, Barcelona, Spain

**Keywords:** Burnout, Work environment, Organizational factors, Perioperative nurses, Leadership, Exhaustion

## Abstract

**Background:**

Knowing the organizational factors that predict burnout in perioperative nurses is paramount for improving the care of patients and promoting nurses’ psychosocial well-being and health.

**Objective:**

To determine the influence of organizational factors of the perioperative nurse's work environment on the three burnout dimensions: emotional exhaustion, despersonalization, and personal accomplishment.

**Method:**

A cross-sectional study was conducted among 136 nurses in a perioperative care unit at a university hospital in Barcelona, Spain. Data were collected using a demographic data form, and the Spanish versions of the Practice Environment Scale of the Nursing Work Index and Maslach Burnout Inventory.

**Results:**

Findings showed emotional exhaustion in 43% (56) of nurses, depersonalization in 21% (28), and reduced personal accomplisment in 53% (69). The degree of general burnout was moderate.

The work environment was considered unfavourable as only one factor of five was favourable (Nursing foundations of quality care). Multiple regression analyses showed three organizational factors were associated with all three dimensions of burnout: “Nurse manager ability, leadership, and support of nurses”; “Staffing and resources adequacy”; and “Nursing foundations of quality care”.

**Conclusions:**

In this study three organizational factors played a significant role in predicting burnout among perioperative nurses. We recommend hospital management implement policies to improve these organizational factors. Promoting positive leadership styles, providing necessary resources, and creating a positive climate in the work environment could increase psychosocial wellbeing and decrease burnout among perioperative nurses.

## 1 INTRODUCTION

Changes in nurses’ working conditions in recent years have shown to influence the working environment and may impact on areas such as job satisfaction, burnout, perceived quality of care, and patient well-being [1, 2]. In Spain, current healthcare budget cuts have made the workplace environment more challenging for nurses in general and also for nurses working in the perioperative area because the workload has increased and surgical waiting lists have grown. As a result, stress levels have risen considerably and may be a factor of burnout among perioperative nurses [3].Perioperative nurses take care of the patient throughout the perioperative process, which covers admission, surgical intervention, and recovery in the post-anaesthesia care unit [4]. The roles of perioperative nurse ensure the quality of perioperative care and surgical patient safety [5]. However, certain stress factors limit nurses’ ability to work to provide best patient care. Several studies have shown that in the perioperative work environment, job stress and burnout are related to factors such as workload, time pressure, patient safety, inadequate communication among team members, feelings of unpreparedness for procedures, and demands for continuous learning [6-9].

Burnout is a syndrome defined by emotional exhaustion that results in depersonalization and decreases in personal work accomplishments [10].The consequences can be negative for the efficiency of the organization, decreasing productivity and the quality of care [11]. Burnout is thus associated with negative outcomes for nurses, inhibiting the development of professional nursing whose aims are to achieve the best results for patients [1].There is no doubt that burnout is a serious issue facing our profession [12, 13].

International studies have documented an association between nurses’ working environment and health outcomes [14, 15]. Research and measurement of the work environment has been recommended as an effective strategy to improve the workplace environment and reduce nurse burnout [16]. This research, which started in the US at Magnet hospitals, has shown that care excellence provides good nursing practices that are associated with positive results for both patients and nurses [17]. Magnet hospitals demonstrate the capacity to attract and retain professionals as they promote quality patient care, safety, interdisciplinary collaboration, positive communication, professional models of care practice, more opportunities for professional development and better practice environments than other hospitals [18]. In contrast, nurses working in environments with negative features are dissatisfied with their job [19], express greater intention of leaving their jobs [20], and are at higher risk of burnout [2]. These feelings have negative consequences on nurses’ mental health and general health and on quality of care for patients [21]. Various instruments have been developed to analyse care environments but that most widely used is the Nursing Work Index (NWI). This tool, which was first developed from research in Magnet hospitals, can measure, assess and compare the nursing practice environment [22]. Two decades after its implementation (2012), within the RN4-Cast (Nursing Care Research Project), several research lines forming part of the study of nurses’ working conditions in relation to patient health outcomes showed deficits in quality of care, job satisfaction and burnout among nurses in intensive care and medical-surgical environments [14].

Spain, along with Greece, is one of the countries with the worst practice environments for nursing care. In addition, it has the lowest nurse/patient ratio and the highest level of burnout [23]. The nurse/patient ratio is 5.4 per 1,000 inhabitants, while in the Organization for Economic Cooperation and Development (OECD) countries the average is 9 [24]. A number of Spanish studies about the working environment have observed high levels of burnout among nurses [25, 26]. However, in a review of the literature review, we found very few data concerning burnout and working environments in nursing practice in the perioperative setting [5, 8]. No studies have been conducted to date regarding perioperative nurses and the characteristics of their working environments in Spain. For this reason, we posed the following three research questions: What are the burnout levels in perioperative nurses? What is the practice environment in the perioperative unit? And which organizational factors explained the burnout dimensions of perioperatives nurses?

The aim of the study was to determine the influence of organizational factors on the perioperative nurses’ working environment on the three burnout dimensions: Emotional exhaustion, depersonalization and personal accomplishment.

## 2 MATERIAL AND METHODS

### 2.1 Description of Study Design, Setting, Population and Sample

We conducted a cross-sectional and analytical study using convenience sampling in a perioperative care unit at a university hospital in Barcelona, Spain. Survey data were collected in 2014. The participants were all staff nurses who had worked for more than one year in perioperative care unit. Nurses on sick leave or on vacation at the time of data collection were excluded. The sample size was calculated based on 95% confidence intervals and a margin of error not exceeding ±5 percent. Questionnaires were distributed to 136 perioperative nurses; 133 (98%) were returned and 130 (95%) were correctly completed. This resulted in a study sample of 130 nurses.

The instruments used in this study were the validated Spanish versions of the Maslach Burnout Inventory (MBI) and the Practice Environment Scale of the Nursing Work Index (PES-NWI)

### 2.2 Maslach Burnout Inventory

The Maslach Burnout Inventory (MBI) [10] has proven reliability and validity [27]. It consists of 22 items (7 point Likert-type items ranging from 0=never, to 6=everyday) and it is structured around three dimensions: a) Emotional Exhaustion (EE), which measures feelings of being emotionally drained and exhausted by one’s work (nine items); b) Depersonalization (D), which represents a lack of feeling and impersonal response towards recipients of one’s service or care treatment (five items); and c) Personal Accomplishment (PA), which measures the feelings of workers to negatively self-evaluate themselves and leads to a diminished sense of competence and success (eight items).The subscales have good internal consistency, ranging from 0.75 to 0.93 [10]. The validity and reliability of the Spanish adapted version MBI was adequate [28]. Cronbach’s alpha values in our study were 0.9 for emotional exhaustion, 0.79 for despersonalization, and 0.71 for personal accomplishment. The items in each subscale were summed. For the emotional exhaustion dimension, scores of 27 or more were considered high, scores ranging from 19-26 were moderate, and scores under 19 were considered low. For the depersonalization dimension, scores equal to or higher than 10 were high, those from 6 to 9 were moderate, and those of less than 6 were low. For the personal accomplishment dimension, scores equal to or lower than 33 indicated a strong sense of low personal accomplishment, those from 34 to 39 were moderate, and those of 40 or more indicated a high sense of lack of achievement. Burnout score was considered high when two or three dimensions had high levels, moderate when two or three had moderate levels or there was one dimension in each level and low when two or three had low levels [29].

### 2.3 Practice Environment Scale-Nursing Work Index (PES-NWI)

The organizational factors in the perioperative environment were measured using the Spanish version of the PES-NWI [30]. This 31-item instrument consists of five organizational factors which asked nurses about their perceptions. The five factors are: 1) “Staffing and resource adequacy” - the current nurse staff to patient ratio levels (four items); 2) “Collegial nurse-physician relations” - teamwork and collaboration between nurses and physician (three items); 3) “Nurse manager ability, leadership and support of nurses” - the nurse managers provided an environment that supported and recognized achievements of nursing staff and showed quality leadership (five items); 4) “Nursing foundations for quality care” - access to continuous training, and nursing standards that are based on a nursing model of care (ten items); and 5) “Nurse participation in hospital affairs” (nine items) - the involvement in policy decisions within the hospital, access to and visibility of senior nurse management, and career development opportunities. This questionnaire used a Likert-type scale where 1 = disagree, 2 = somewhat disagree, 3 = somewhat agree, and 4 = agree. Classification regarding the practice environment is agreed according to the PES-NWI: Favourable when there are 4 or 5 factors with a score > 2.5; mixed when there are 2 or 3 factors with a score >2.5; and unfavourable when there is one or no factor >2.5. The psychometric tests determined that the PES-NWI had a stable factor structure, high internal consistency, and adequate reliability. The original scale was developed by Lake 2002 [31] Cronbach's coefficient of the PES-NWI was 0.82, and the coefficient for each subscale ranged from 0.71 to 0.84. The Spanish version of Cronbach’s alpha was 0.90 (95%IC:0.87-0.93) for the general scale and the coefficients for each of the five PES-NWI factors were between 0.73 and 0.81.

### 2.4 Statistics Analysis

Descriptive statistics were used to characterize the sample (frequencies and percentages for the categorical variables and measures of main trend and dispersion for the quantitative variables). Mean scores obtained for the MBI dimensions were transformed into moderate-high level burnout and low level burnout for each nurse. The relationship between burnout and explanatory variables was identified using the Chi-squared test; when hypotheses for this test were not confirmed (at least 80% of the cells in a contingency Table must be above 5) we used Fisher's Exact Test. Finally, multivariate linear regression was used to determine the relationship between the NWI-PES factors and burnout dimensions, adjusting for the explanatory variables. The statistical package SPSS 22.0 (IBM SPSS, Spain) was used for data analysis.

## 3 RESULTS

Table **1** shows the participants’ socio-demographic and professional characteristics. A total of 130 surveys were included (95.6%), representing a high degree of participation. The nurses’ mean age was 43.5 years (SD = 11.9) and 91.5% (119) were women. The average number of years of nursing experience was 21 (SD = 12.13) and the average number of years of experience in the area was 14 (SD=11.14). Seventy-four percent of participants worked in the operating room area and 58.5% worked the morning shift. Most (83.1%) had permanent work contracts. A total of 98.5% of those surveyed had specialized training (Master or Postgraduate courses) (Table **1**).

### 3.1 Nurse Burnout

Overall results of this study indicate the nurses had moderate-high levels of burnout according to the average scores obtained in the three-burnout dimensions.

A moderate-high level was 43% (n=53) for emotional exhaustion; 21.5% (n=28) for despersonalization; and 53% (n=69) for personal accomplishment. A total of 41% (n=53) of nurses showed moderate-high-high burnout in global terms, and 59% (n=77) showed low burnout. Table **2** shows the mean and standard deviations for each burnout dimension (Table **2**).

### 3.2 Organizational Factors

The perception of the perioperative environment was unfavorable among nurses because only one factor (Nurse foundations for quality of care) reached the most favorable mean scores 2.65 (SD 0.52; min= 1.20, max= 3.8).The factors “Nurse participation in hospital affairs”, “Staffing and resource adequacy”, “Nurse manager ability, leadership, and support of nurses” and “Collegial nurse-physician relations” showed low mean scores: 1.96 (SD 0.43; min= 1.11, max= 3.44); 2.12 (SD 0.65; min= 1, max =3.75); and 2.27 (SD 0.67; min=1.00, max= 4) and 2.47 (SD 0.68; min=1.00, max= 4), respectively were unfavourable.

### 3.3 Relationship Between MBI Dimensions and Demographic and Professional Characteristics

Regarding demographic and professional characteristics and MBI dimensions, a bivariate analysis showed that emotional exhaustion was related to age (as age increases, so does degree of burnout); type of service (highest levels of emotional exhaustion were observed in nurses working in pre and post-surgery areas; the type of contract (higher levels of emotional exhaustion were documented among nurses on permanent contracts). Emotional exhaustion was also related to work experience (the more experience, the greater the emotional exhaustion). Civil status (being married), working shift (mornings) and the type of contract (permanent) were related to higher personal accomplishment. However, there was no significant relationship between despersonalization and these factors (Table **3**).

Despersonalization was negatively related to the factor “Nurse manager ability, leadership, and support of nurses" (r-1.5; p=0.047). When this factor increased, despersonalization decreased. Lastly, the personal accomplishment was positively related to “Nursing foundations for quality care” (r 4.14; p = 0.014). If this factor increased, so did the degree of personal accomplishment. None of the other organizational factors were associated with personal accomplishment in the adjusted models.

In the perioperative area, inadequate staffing and lack of leadership and support for nurses increased the emotional exhaustion and despersonalization. The relationship between personal accomplishment and Nursing foundations for quality care was statistically significant (Table **4**).

Models adjusted by nurses’ characteristics: Sex, civil status, service, service (surgical/non-surgical), shift, type of contract, sick leave, years of experience and years of experience in current position.

Following the findings from the bivariate analysis, stepwise multiple regression analysis was undertaken to determine the association of age, sex, civil status, service operating room /nonsurgical (pre-post-surgical), working shift, and type of contract, sick leave and years of experience in the current position but only the organizational factors of practice environment, had a statistically significant relationship.

3.4

Relationship Between Burnout Dimensions and NWI-PES Factors

We carried out a multivariate logistic regression analysis and examined the relationship between the organizational factors in the practice environment (Staffing and resource adequacy, Collegial nurse-physician relations, Nurse manager ability, leadership and support of nurses, Nursing foundations for quality care, and Nurse participation in hospital affairs) and burnout dimensions (emotional exhaustion, despersonalization and personal accomplishment) .

Table **4** provides results from adjusted linear regression models for the three burnout outcome variables. Only three organizational factors had a statistically significant relationship. The organizational factors, “Staffing and resource adequacy” (r -3.46; p=0.013) and “Nurse manager ability, leadership and support of nurses”(r -3.90; p = 0.023) were negatively related to emotional exhaustion dimension in the models adjusted to nurses’ characteristics (sex, civil status, service operating room /nonsurgical (pre-post-surgical), working shift, and type of contract, sick leave and years of experience in the current position).

## 4 DISCUSSION

In this study we measured the organizational factors of the work environment and their association with the three dimensions of burnout in nurses in the perioperative area. Our study findings revealed Emotional Exhaustion (EE) in 43% of nurses, Depersonalization (D) in 21%, and Personal Accomplishment (PA) in 53%. The degree of general burnout was moderate. Nearly half of the nurses reported moderate-high burnout in emotional exhaustion, less than one-third had moderate to high burnout in despersonalization, and more than half showed low burnout in personal accomplishment. Some Spanish studies show a similar or even higher burnout [26, 29, 32], reflecting a high incidence of burnout among nurses in general in Spain. These results are consistent with many international studies with similar conditions of staff shortages and poor practice environments [14, 20]. In some of these studies, such as several from Turkey and Greece, burnout was even higher than in Spain. Several studies have shown how budget cuts have direct negative effects on staffing and practice environments of health professionals [33, 34].

In relation to the perception of the perioperative nurses’ environment work in our study, most nurses perceived their work environment as unfavourable for four of the five organizational factors: “Participation in hospital affairs”, “Nurse Managers’ ability, leadership and support of the nurses”, “Nurse - physician relation”, and “Staffing and resources adequacy”. Only one factor, “Nursing foundation quality of care”, was favourable. These results support findings from other studies conductued in hospitals with low staffing levels in areas where nurses care for critically ill patients who have complex conditions and needs [35, 36]. The most important finding in our study was that three organizational factors were associated with the dimensions of burnout: “Nurse manager ability, leadership, and support of nurses”; “Staffing and resources adequacy”; and “Nursing foundations of quality care”. The factor leadership and support for nurses were related to emotional exhaustion and depersonalization. Low leadership support for nurses has previously been shown to have a strong effect on nursing dissatisfaction and burnout [37]. Lack of recognition and unfair treatment is emotionally exhausting, possibly leading to a deep sense of cynicism about the work and increasing professional’s vulnerability to burnout [21]. The role of perioperative nurse leaders in the development of developing nurses’ burnout, should therefore be addressed [38]. These relationships are consistent with those of other studies which found that good nurse leadership can prevent emotional exhaustion among nurses [39, 40].

Shortages of staff and resources are a major concern of nurses as they are unable to meet all the demands of their work. Fewer nurses obviously increases the workload and has been clearly related to a greater risk of burnout [14, 41]. Shortages are likely of particular note in operating rooms and recovery rooms - in some places in our setting there are now only two nurses where there had previously been three nurses - as complexity has increased considerably in recent years due to advances in technology and greater numbers of aged patients with complex comorbidity [27].We did not find any evidence regarding standardized measures of nurse staffing for the surgical area and each hospital has its own staffing criteria to schedule the number of professionals needed for each intervention [42]. The RN4Cast focused on medical-surgical units and intensive care units, but to our knowledge, no studies have been performed to determine the number of surgical nurses needed in perioperative areas. Besides, there are no guidelines in Europe from any organization such as NICE (National Institute for Health and Care Excellence) or EORNA (European Operating Room Nurses Association) that have developed recommendations in this topic.

As concerns personal accomplishment, in our study this factor was related to “Nursing foundations and quality of care”. Despite the level of burnout among perioperative nurses and the unfavourable work environment, the nurses reported that their standard of care and clinical competence were high. This findings supports the observation of Lashinger & Leiter that nurses may compensate for the negative aspects of their work environment with their professional competency [43].

Analysis of the association between the presence of burnout and demographic and professional characteristics such as age, type of contract, job type and years in current job showed burnout was higher in older nurses who worked in postoperative services and had a permanent contract. In the multivariate analysis, however, these differences were not statistically significant. These results are consistent with observations in the literature that the burnout is primarily related to the nurse working environment and organizational factors, and that personal factors and demographic factors are secondary causes [44, 45].

Finally, several studies highlight the importance of considering job satisfaction and work engagement as environmental variables within health organizations so as to prevent burnout and job-related distress and to promote wellbeing of workers [46-48]. In the workplace included in the present study it is necessary to consider the impact that dimensions and indicators of organizational health have on the quality of work and on the perception of well-being of workers, such as the openness to the external environment and technological and cultural innovation [49].

### 4.1 Limitations

The study has a number of limitations. First, in view of the cross-sectional design, our results should be interpreted with caution. Second, the study was performed in a single centre, but as this is a comprehensive general public hospital and all the perioperative nurses participated in the survey we consider it is is representative of the current climate in perioperative nursing in Spain.

### 4.2 Managerial Implications

This study provides evidence of the impact that the perioperative working environment may have on nurses’ burnout. To improve job satisfaction among perioperative nurses and consequently that of surgical patients, hospital administrators should address factors linked to the job (such as physical and emotional aspects), factors linked to the organizational context, and factors connected to the openness to the external environment and technological and cultural innovation, so as [49].

In addition they should ensure standards of safety for their patients by evaluating how care delivery is provided in their organization and changing nurses’ working conditions. Quality of nursing practice and quality of patient care by perioperative nurse leaders must be encouraged. Besides, nursing managers and staff training in effective strategies is essential to decrease and prevent burnout. Studies such us can contribute in the development of strategies would improve perioperative nurses’ efficiency and the quality of care.

Further research is required to corroborate our results and determine whether patient outcomes are affected by factors related to the perioperative nursing environment.

## CONCLUSIONS

Our results indicate that adequate staffing, leadership, and support for nurses and nursing foundations for quality care are paramount to avoid burnout in perioperative nurses.

Determining factors leading to burnout in perioperative nurses is essential to implement strategies to improve the care of surgical patients and promote psychosocial well-being and health of nurses in the perioperative setting.

## Figures and Tables

**Table 1:** Demographic and professional characteristics of the study sample (N=130).

**Socio-Demographics Variables**	**n**	**%**
Gender		
Female	119	91.5
Male	11	8.5
Age		
21-30	26	20.0
31-40	32	24.6
41-50	25	19.2
51-65	47	36. 2
Civil status		
Married	75	57.7
Single	45	34.6
Widowed	4	3.1
Divorced	6	4.6
Nursing Education^1^		
Diploma degree	18	13.8
Master’s degree	41	31.5
Postgraduate education	87	66.9
Service		
Operating Room	96	73.8
Others^2^	34	26.2
Shift		
Morning	76	58.5
Afternoon	23	17.7
Night	20	15.4
Others	11	8.5
Type of contract		
Permanent	108	83.1
Temporary	22	16.9
-	**M**	**SD**
Age	43.5	11.90
Years worked as a nurse	21.6	12.13
Years worked in current position	14.0	11.14

1. Total more than 100 because they are not mutually exclusive categories 2. Reception room surgical patient and recovery room.

**Table 2:** Nurse burnout scores (distribution in low or moderate-high/high levels, mean and standard deviation) (N=130).

	**Emotional Exhaustion**		**Despersonalization**		**Personal** **Accomplishment**		**Global**	
	**n**	**%**	**N**	**%**	**n**	**%**	**N**	**%**
**Low**	74	56.9	102	78.5	61	46.9	77	59.2
**Moderate-high**	56	43.1	28	21.5	69	53.1	53	40.8
	**M**	**(SD)**	**M**	**(SD)**	**M**	**(SD)**	**M**	**(SD)**
	17,9	(10,2)	3,1	(3,7)	37,7	(7,7)	58,7	(12,3)

**Table 3:** Levels of significance (P-values) of the statistical tests of the bivariate analysis for explanatory variables against MBI dimensions (N=130).

Socio-Demographics Variables	Emotional Exhaustion	Depersonalization	Personal Accomplishment
Sex^a^	0.757	0.250	0.113
Age^b^	0.002	0.636	0.028
Civil status^a^	0.224	0.727	0.001
Service^b^	0.006	0.236	0.819
Shifts^b^	0.334	0.458	0.041
Type of contract^a^	0.002	0.783	0.010
Sick leave in previous 2 years^b^	0.069	0.503	0.613
Years of work experience^c^	0.001	0.419	0.074

^a^ Bivariate analysis by means of the Fischer Exact Test **^b^** Bivariate analysis by means of the Chi-squared Test ^c^ Bivariate analysis by means of a mean comparision

**Table 4:** Multivariate analysis. Adjusted regression coefficient indicating the effect of Organizational Factors of work environment nurse on Burnout in nurses. (N=130).

**Organizacional Factors**	**Emotional Exhaustion**	**Depersonalization**	**Personal Accomplishment**
	Adjusted B (SD)	Adjusted B (SD)	Adjusted B (SD)
**Staffing and Resource Adequacy**	-3.46 (1.37)* p = 0.013	-0.27 (0.61) p = 0.654	-0.16 (1.20) p = 0.895
**Collegial Nurse-Physician Relations**	-0.54 (1.3) p = 0.679	-0.53 (0.58) p = 0.360	0.12 (1.15) p = 0.917
**Nurse Manager Ability, Leadership, and Support of Nurses**	-3.90 (1.69)* p = 0.023	-1.5 (0.75)* p = 0.047	1.72 (1.48) p = 0.247
**Nursing Foundations for Quality Care**	-0.23 (1.90) p = 0.902	-1.4 (0.84) p = 0.097	4.14 (1.66)* p = 0.014
**Nurse Participation in Hospital Affairs.**	-2.25 (2.64) p = 0.397	2.22 (1.17) p = 0.060	-1.45 (2.31) p = 0.531

*p < 0•05;
